# Cardiorespiratory fitness response to endurance training in athletes post-COVID-19 compared to unaffected athletes

**DOI:** 10.17159/2078-516X/2024/v36i1a18872

**Published:** 2024-01-15

**Authors:** CA Haley, G Torres, B Olivier, H Van Aswegen

**Affiliations:** 1 Department of Physiotherapy, School of Therapeutic Sciences, Faculty of Health Sciences, University of the Witwatersrand, Johannesburg, South Africa; 2 Department of Exercise Science and Sports Medicine, School of Therapeutic Sciences, Faculty of Health Sciences, University of the Witwatersrand, Johannesburg, South Africa; 3 Centre for Healthy Living Research, Oxford Institute of Allied Health Research, Department of Sport, Health Sciences and Social Work, Oxford Brookes University, Oxford, United Kingdom

**Keywords:** cardiopulmonary exercise test, coronavirus, exercise intolerance

## Abstract

**Background:**

Endurance sports primarily attract recreational athletes over 35 years, who impose an exceptionally rigorous and sustained demand on their cardiorespiratory systems.

**Objectives:**

This study aimed to determine the influence of COVID-19 on the cardiovascular, pulmonary, and skeletal muscle function of endurance athletes with exercise intolerance. Secondly, it aimed to compare the exercise response of endurance athletes post-COVID-19 to those unaffected using cardiopulmonary exercise test-related variables.

**Methods:**

This is a prospective observational cohort study of endurance athletes. An exposure group with protracted exercise tolerance underwent a resting lung function test and maximal cardiopulmonary exercise test. These were repeated after eight weeks of endurance training and compared to the published reference values and a control group of athletes unaffected by COVID-19.

**Results:**

The post-COVID-19 exposure group (n=57), mean age 44.5±8.1years showed a poorer ventilatory threshold (p=0.004), and workload (p=0.05), with higher respiratory exchange ratio (p=0.05) than the control group (n=34), mean age 41.8±7.7 years. Maximal inspiratory pressure improved at follow-up in the COVID-19 group compared to the controls (p=0.03). Increased odds of pulmonary and skeletal muscle limitation to aerobic capacity were found in the COVID-19 group. The COVID-19 group responded positively to endurance training with improved VO_2_peak (p=0.005), maximal inspiratory pressure (p=0.04), oxygenpulse (p=0.02), and maximal workload (p<0.001).

**Conclusion:**

COVID-19 has led to pulmonary and extrapulmonary limitations to exercise capacity. Tailored intensity and duration of physical activity are vital after COVID-19 to restore skeletal muscle health. This multidisciplinary rehabilitation approach will optimise the resumption of participation in long-distance events.

The highly infectious novel coronavirus disease of 2019 (COVID-19) caused by the severe acute respiratory coronavirus 2 (SARS-CoV-2) caused a worldwide pandemic. The expression of this disease ranged from completely asymptomatic, through a wide variety of symptoms classed as mild, moderate, or severe illness, to death.^[1]^ The pathophysiology of COVID-19 can lead to persistent symptoms, predominantly dyspnoea and fatigue^[2]^, which are associated with poor exercise tolerance.^[3]^ Scheer et al.^[4]^ have shown that average marathon times declined worldwide during the pandemic. However, the impact of COVID-19 on athletic ability remains equivocal as some studies found a decrease in cardiorespiratory fitness (CRF),^[5]^ while others found no difference.^[6]^ Physical activity has numerous benefits, including enhanced immune response, which mitigates the risk of post-COVID cardiopulmonary events.^[7]^ Additionally, exercise fosters mental resilience, muscular endurance, and emotional well-being.^[8]^ Therefore, the resumption of sport and competition is paramount for athlete health.

Endurance sport typically involves physical activity for a prolonged duration, such as marathon running and long-distance cycling.^[4,9]^ Endurance athletes impose an exceptionally rigorous and sustained demand on their cardiorespiratory system throughout training and competitive events.^[7]^ Respiratory muscles fatigue over time during endurance exercise. Therefore, pulmonary limitations will have a greater impact than in power sport athletes.^[10]^ The work of breathing, measured by maximal inspiratory pressure (MIP), relates to respiratory muscle strength and elasticity of the lung and chest wall,^[10]^ both of which are adversely affected by COVID-19. This sport primarily attracts recreational athletes over the age of 35, who often participate at a competitive level.^[11]^ For COVID-19 convalescent athletes, regaining adequate levels of CRF is crucial as a small decrease in performance capacity can have a marked effect on an athlete’s goals.

Cardiopulmonary exercise testing (CPET) is the gold standard measurement of CRF^[3]^ and objectively detects limitations to exercise capacity by the pulmonary, cardiovascular, or skeletal muscle systems.^[5,12]^ It is especially beneficial for assessing athletes with a protracted return to the sport due to persistent exertional symptoms without direct viral organ damage.^[13]^ The cardiopulmonary exercise test measures the maximum oxygen uptake during dynamic exercise (VO_2_peak), directly evaluating an individual’s CRF.^[6]^ Endurance athletes are expected to exceed the published thresholds for CPET parameters applicable to healthy adults.^[14]^ Failing which, concerns regarding possible impairments arise.^[15]^ Limited research exists on long-term limitations to exercise capacity in endurance athletes^[5]^ and the cardiorespiratory response as these athletes resume endurance training. Insight into the mechanisms impacting athletic performance after recovering from the acute phase of the disease will assist clinicians in preparing athletes for the successful resumption of competition.^[10]^ This study aimed to determine the influence of COVID-19 on the cardiovascular, pulmonary, and skeletal muscle function of endurance athletes using CPET-related variables. Secondly, it aimed to compare the exercise adaptation of endurance athletes post-COVID-19 to a control group of athletes unaffected by COVID-19. In this post-pandemic era, the imperative of regular physical activity cannot be overstated; thus, identifying persistent limitations to exercise capacity is vital.

## Methods

### Study design

This is a prospective observational cohort study. The STROBE Statement is a checklist of items that should be included in cohort studies reports, which was followed accordingly.^[16]^ The study occurred in Johannesburg, the most populous city in South Africa (1450 m above sea level). Participants were recruited between 15 December 2021 and 1 October 2022. The initial testing and simultaneous data collection commenced on 8 March 2022, and eight-week follow-up testing terminated on 28 October 2022.

### Participants

**Fig. 1:**
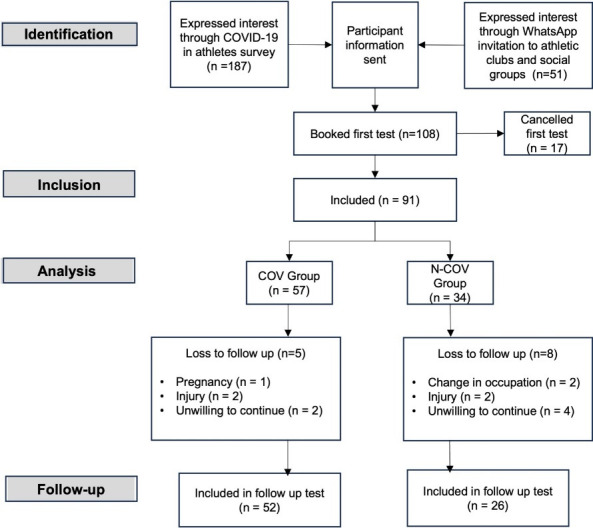
Process of participant recruitment

Social media advertisements were approved by athletic clubs and posted on their Facebook pages (n=25) and WhatsApp groups (n=18). They included a clickable link to more information about the study according to ethical principles. Long-distance runners and cyclists who responded to the advertisements were eligible for participation. It is assumed that participants responded to the advertisement randomly, reducing potential selection bias and confounding variables such as comorbidities. The process of recruitment of participants is displayed in Figure 1.

The inclusion criteria for both study groups were adult endurance athletes (marathon minimum age is 18 years), who were eligible if they engaged in cardiovascular exercise for three or more hours per week.^[11]^ COVID-19 convalescent athletes who had persistent symptoms affecting their exercise capacity were allocated to the exposure group (COVID-19), and COVID-negative or those who were unaffected by the infection and had successfully returned to their pre-illness fitness levels were allocated to the control group (N-COVID-19). A minimum time since infection of 18 days and the ability to run for 30 min was required. Those with comorbidities or severe disease needed to consult a sports physician before participating.

Exclusion criteria were: athletes with ICU-acquired weakness, lack of signed informed consent, not being proficient in English, residing outside of Gauteng, and published contraindications to CPET.^[17]^

### Testing procedure

The research team developed self-administered web-based questionnaires for the exposure and control group using the Research Electronic Data Capture (REDCap) platform. These were disseminated via email or WhatsApp to potential participants and were completed before testing to establish the subjective examination. They included demographic and sport-specific data; physical activity level determined by the number of hours per week the athlete exercised; and questions regarding the severity of symptoms directed to the exposure group and classified according to the NIH clinical spectrum.^[1]^

Eligible participants were sent a link to an online calendar to book their CPET. At each visit, all participants were assessed to determine their medical history and management, and the presence of known contra-indications to CPET. The time interval from infection to first test and between tests was recorded. Age, sex, weight, height, BMI, and blood pressure were collected before the lung function test and CPET were performed. Data collection was standardised by using consistent measurement tools in the same setting.

### Lung function

The primary researcher assessed resting lung function in standing using spirometry. The Vyaire Medical CPET BxB Sentry Suite^™^ V2.21 software was used to record data during the test. Measurements are described in Table 1. Maximal inspiratory pressure was assessed in standing using the POWERBreath K3 device. The best of three attempts with a less than 5% difference was recorded according to the American Thoracic Society protocol.

### Cardiopulmonary exercise testing (CPET)

CPET was conducted using a treadmill test rather than a cycle ergometer as it gives higher peak oxygen uptake results to athletes.^[18]^ A General Electric Healthcare CASE^™^ exercise testing system, version 6.7, Milwaukee, USA, was programmed according to the Bruce protocol suitable for athletes. ^[18]^ Throughout the test, continuous monitoring of heart rate was done using a 12-lead ECG, and oxygen saturation (SpO_2_) using a pulse oximeter. Participants were blinded to test data while performing the treadmill test. Rating of perceived exertion (RPE) on the 20-point Borg scale was collected at each stage^[19]^ and the reason for termination was noted. The Vyaire Medical CPET BxB Sentry Suite^™^ V2.21 software was used to record data during the test. A gas analyser allowed the analysis of inspired and expired gases every five seconds.^[17]^ The participants were verbally encouraged to give their best effort and keep going until volitional termination due to exhaustion or provocation of symptoms, with the participant rating above 18 on the RPE scale. Respiratory exchange ratio (RER) >1.1 is defined as a maximal test. The results were displayed in the Wasserman “nine-panel plot” format.^[12]^ Physiological parameters that were calculated by the software are described in Table 1.

### Statistical analysis

The sample size calculation was based on the difference in VO_2_peak in the COVID-19 vs N-COVID-19 groups from the study by Ladlow et al.^[5]^ with a confidence interval of 0.05 and a statistical power of 0.8 selected. We added 10% for each confounding factor: age, sex, stage of disease, and loss to follow-up. Therefore, a sample size n=50 was required. We aimed for a 2:1 ratio of symptomatic to control/recovered.

Data were collected and managed using MS Excel and imported into the Statistical Package for the Social Sciences software, IBM SPSS Statistics for Mac, Version: 28.0.2.0 (142), which was used for all analyses. Descriptive characteristics were used to summarise the data. The normality of the distribution of continuous variables was assessed with the Shapiro-Wilk test. Continuous variables are expressed as means and standard deviations for normally distributed data, medians and interquartile range for skewed data. Categorical variables are expressed as percentages and frequencies. Normative values have been categorised by sex and age groups in decades, according to the FRIEND Registry.^[15]^ Independent t-tests and paired t-tests were used to compare normally distributed parametric variables, and the Mann-Witney U test and Wilcoxon Signed Rank for skewed, nonparametric continuous data. Chi-square or Fisher’s exact tests were used to compare categorical data. Odds ratios determined the likelihood of limitations to exercise capacity after having COVID-19. A p-value of <0.05 determined statistical significance. Missing data of loss-to-follow-up participants was only excluded in comparing the CRF response to endurance training between the COVID-19 and N-COVID-19 groups. No imputation was used for the missing data.

### Data availability

Data will be made available upon request and after considering the intentions of use, ethical clearance, and appropriateness of available data. The corresponding author may be contacted in this regard.

### Ethical considerations

Unconditional ethical clearance was provided by the University of the Witwatersrand (25 October 2021). The study procedures adhered to the principles outlined in the Declaration of Helsinki, the South African Guidelines for Good Clinical Practice, and the Medical Research Council. Each participant signed informed consent before the first test. It ensured confidentiality (including protection of personal information), anonymity, permission to withdraw from the study at any point, and data sharing.

## Results

The first assessment was attended by: the COVID-19 group (n=57), mean age 44.5±8.1 years, and the N-COVID-19 control group (n=34), mean age 41.8±7.7 years.

No significant differences in population demographics between COVID-19 and NCOVID-19 groups existed (Table 2).

During the pre-test assessment, the researcher found that two participants had been diagnosed with myocarditis post-COVID-19. One was prescribed beta-blockers and cleared for exercise, while the other confirmed that the condition had resolved. All participants reached a maximal heart rate of at least 85% maximum, predicted at the termination of both tests. In the first test, systolic blood pressure >250mmHg was the reason for termination in n=1 (1.8%) in the COVID-19 group and was referred for further investigation. Hypotension was found in one participant, and paraesthesia was found in two participants. Dyspnoea terminated the test in n=24 (42%), three of whom had been suffering from anxiety. Volitional exhaustion terminated all the tests in the N-COVID-19 group, with two participants complaining of dizziness at maximal exertion. No adverse events occurred during the maximal exercise tests.

Baseline CPET found a higher respiratory exchange ratio at maximal exercise (p=0.05, CI=0–0.05, d=0.43), and strong evidence of a lower ventilatory threshold (p=0.004, CI=−11.42– −2.29, d=−0.65) and lower maximal workload (p=0.05, CI=0–1.10, d=0.71) in the COVID-19 group. No differences in lung function measures were noted when comparing the two groups.

On follow-up, dyspnoea terminated the test in n=17 (33%) in the COVID-19 group. When comparing the exercise response to endurance training (Table 3) there is strong evidence of a process of recovery shown by improved MIP in the COVID-19 group versus a decline in the NCOVID-19 group (p=0.03, CI=0.94–16.22, d=0.54). The difference in maxRER in the COVID-19 group declined, whereas it increased in the N-COVID-19 group (p=0.05, CI=0.03–0.05, d=0.58).

When comparing the exercise response in the COVID-19 group (baseline versus follow-up tests), there is strong evidence of a process of recovery over time shown by improved VO_2_peak (p=0.005, d=0.–41) and maximal workload (p<0.001, d=0.5), moderate improvement in MIP (p=0.04, d=−0.38), and weak evidence of improved O_2_-pulse (p=0.03, d=−0.31), (Supplementary Table 1). No meaningful differences were found in the N-COVID-19 group (Supplementary Table 2).

There is considerable heterogeneity in the range of aerobic capacity of the sample (Supplementary Figure 1). Table 4 displays the odds of pulmonary and extrapulmonary limitations to exercise capacity following COVID-19. There are higher odds of exercise-induced hypoxia post-COVID-19 (p=0.05, OR=7.02, CI=0.86–57.53). The odds ratio of abnormal MIP post-COVID-19 is high, although not showing statistical significance (p=0.07, OR=3.19, CI=1.15–12.2). Similarly, there are higher odds of pulmonary limitations after COVID-19 implied by combining MIP with VE/MVV (p=0.07, OR=4.27, CI=0.89–20.39).

These results may have clinical relevance. Baseline testing of VT suggested skeletal muscle oxygen extraction limitations in the COVID-19 group up to nine months post-illness (p=0.03, OR=5.21, CI=1.11–24.56).

## Discussion

This study provides insight into protracted exercise intolerance following COVID-19 in an athlete population with a high volume of cardiovascular exercise, predominantly of masters-age. VO_2_peak of less than 85% of the predicted value indicates functional cardiorespiratory limitation to exercise capacity in the general population,^[5]^ and in athletes, less than 100% warrants further clinical investigation.^[15]^ Our findings suggest that the athletes had poorer maxRER, VO_2_ at VT, and maximal workload during CPET testing for an average of six months post-COVID-19, when compared to matched athletes. The minimal differences in lung function between groups suggest this cohort’s limitations are not primarily pulmonary.

In our study there is strong evidence of skeletal muscle oxygen extraction limitations in the COVID-19 group, as indicated by the low VT. For endurance athletes, VT is a better predictor of endurance capacity as it relates to skeletal muscle capacity,^[5,15]^ and should typically reach 70–80% of VO_2_peak.[18] The mean time from acute illness to the first test in this study was six months, indicating a prolonged adverse effect on the skeletal muscle system. Fatigue and muscle weakness are common after the onset of COVID-19, potentially accelerating the transition to anaerobic metabolism (as evidenced by a decrease in VT).^[13]^ This stimulates the metaboergoreflex causing an increased VE/VCO_2_ slope.^[3]^ Decreased cardiac output may be the cause; however, it is more likely in our study to be due to impaired mitochondrial function.^[8]^ Physical activity at low intensity restores mitochondrial health,^[8]^ and athlete training programs must consider the prolonged healing time required to achieve this.

In a study investigating patients with exercise intolerance post-COVID-19, a maximum invasive CPET was performed and found that impaired peripheral oxygen extraction and hyperventilation limited exercise capacity more than cardiac abnormalities.^[13]^ Heterogeneity exists in the published literature regarding the primary limitation to aerobic capacity, with reduced O_2_-pulse cited as a potential mechanism.^[2]^ In our study, O_2_-pulse improved at follow-up in the COVID-19 group, consistent with another study,^[20]^ however, athletes with persistently low O_2_-pulse should not be overlooked. There were 23% of the athletes in the COVID-19 group that had elevated VE/VCO_2_ slopes, as opposed to the 18% in the N-COVID-19 group. Considering the positive y-intercept of the VE/VCO_2_ slope in the COVID-19 group, this may be clinically relevant and indicate pulmonary rather than cardiac involvement.

Pulmonary limitations cause impaired perfusion, effectively detected by CPET.^[5]^ Our study results agree with Moulson et al. ^[20],^ who found that among individuals with low breathing reserve, 50% had a true pulmonary limitation. In comparison, 38% had normal resting lung function and supranormal fitness (VO_2_peak>120%), indicating a normal physiological response in well-trained athletes. Further studies assessing aerobic capacity should consider low MIP with abnormal breathing reserve as an indicator of pulmonary limitation. The respiratory system can limit exercise capacity by four mechanisms: increased work of breathing, exercise-induced hypoxemia, respiratory muscle fatigue, or dyspnoea.^[10]^ When comparing the exercise response of endurance athletes, this study found minimal improvement in the resting lung capacity of the N-COVID-19 group. In contrast, the COVID-19 group showed marked improvement in MIP, indicating recovery of respiratory muscle strength.^[21]^ This implies the immediate need for respiratory rehabilitation from the acute phase of the illness, as these results indicate persistent pulmonary limitations more than six months post-COVID-19. Respiratory muscle training may be valuable in this population.

### Limitations

Convenience sampling can lead to selection bias in the control group. However, one assumes that responses to advertisements were random. Participants would have required access to social media. Although pre-illness lung function and CPET data were unavailable, comparison with controls indicated that the difference was likely due to COVID-19 infection. The control group was also exposed to the environmental and psychological impact of the pandemic and related restrictions. These global effects were unavoidable and potentially reduced exercise capacity in the control group; therefore, they may have created a negative bias of the results of the control group.^[4]^ Illness dates are subject to recall bias. The athletes in this study may have been infected with a mixture of different virus strains. The results of this study are generalisable to endurance athletes in a predominantly masters-age group. The broad variance in results reduced statistical significance, and a larger sample size may be required in further studies. These results could be valuable when combined in a systematic review and meta-analysis. The strengths of the study include the demographic characteristics closely matching that of marathon runners worldwide, which fills a gap in the literature. Both exposure and control groups had follow-up tests to account for retraining. This is one of the first studies to describe pulmonary limitations to exercise capacity based on abnormal MIP with low breathing reserve. Future studies investigating pulmonary rehabilitation, including respiratory muscle training is recommended. Physical activity aiming to restore mitochondrial health warrants further research in athletes with post-viral fatigue. Further research is recommended to determine whether targeted interventions will facilitate the return to pre-illness CRF. The long-term effects of COVID-19 to date warrant investigation.

## Conclusion

This study aimed to determine the influence of COVID-19 on the cardiovascular, pulmonary, and skeletal muscle function of endurance athletes with exercise intolerance and compare their exercise response to a control group. Our findings suggest impaired oxygen extraction by the skeletal muscle reduces aerobic capacity in endurance athletes after COVID-19. Pulmonary limitations were present in the post-COVID group, including weakened respiratory muscle strength. Resisting fatigue is imperative when exercising for a prolonged duration. Thus, both pulmonary and extrapulmonary limitations need to be managed. This underscores the need for rehabilitation tailored to recovering pulmonary or skeletal muscles in athletes after respiratory diseases. A multidisciplinary approach is recommended to restore athlete health and their ability to achieve their athletic goals.

## Supplementary Information



## Figures and Tables

**Table 1:** Description of the physiological parameters measured during lung function and CPET

Measurement	Description

**Lung function**	

FVC (l)	Forced vital capacity
FEV_1_ (l)	Forced expiratory volume in one second
PEF (l·min^−1^)	Peak expiratory flow
MVV (l·min^−1^)	Maximum voluntary ventilation: estimated by multiplying FEV_1_ by 35^[18]^

**Cardiopulmonary exercise testing (CPET)**	

VO_2_peak (ml·kg·min^−1^)	Maximum volume of oxygen in millilitres consumed per minute per kilogram of body weight
VE (l·min^−1^)	Minute Ventilation: The litres per minute that is inspired and expired
VE/VCO_2_ slope	The volume of CO_2_ expelled per litre of air ventilated. Indicative of ventilatory efficiency. An increase in slope indicates increased dead space ventilation in the lung, pulmonary vascular disease or pulmonary limiting disease.
Y-intercept on VE/VCO_2_ slope (l·min^−1^)	A negative y-intercept is indicative of cardiac limitation
VT (ml·min^−1^)	Ventilatory threshold = The point at which VCO_2_ deviated from the linear relationship with VO_2_. Indicative that oxygen-independent energy systems are starting to contribute relatively more to energy production with a slight increase in blood lactate levels above resting levels.
HR (bmp)	Heart rate (beats per minute)
SpO_2_ (%)	Peripheral capillary oxygen saturation. Drops by 5% in healthy adults and 10% in athletes during maximum exercise.
Relative workload (W·kg^−1^)	Power (W) divided by body weight
VE/MVV (%)	Dyspnoea index: the ratio of peak minute ventilation to maximum voluntary ventilation. Indicates respiratory limitation if it approximates 100%. Breathing reserve = 1-VE/MV
Peak O_2_-pulse (ml·bpm^−1^)	Oxygen pulse calculated by VO_2_peak/HRmax. It is a surrogate measure of left ventricular stroke volume.^[17]^

**Table 2:** Descriptive characteristics of participants: COVID-19 vs N-COVID-19 groups

Parameter	COVID-19 (n=57)	N-COVID-19 (n=34)	p value	95% CI	Effect size

**Male sex n (%)**	36 (63%)	25 (74%)	0.25		

**Age groups (years) n (%)**					
21–29	2 (3.5%)	1 (2.9%)	1.00		
30–39	12 (21%)	13 (38%)	0.08		
40–49	30 (53%)	15 (44%)	0.43		
50–59	11 (19%)	4 (12%)	0.40		
60–65	2 (3.5%)	0	0.53		

**Weight (kg)**	73.4±13.2	74.8±12.9	0.62		

**Height (cm)**	174.2±9.2	175.9±10.1	0.40		

**BMI (kg/m** **^2^** **)**	24.1±2.9	24.0±2.3	0.84		

**Level n (%)**					
Recreational	22 (39%)	14 (41%)	0.81		
Competitive	26 (46%)	18 (53%)	0.50		
Elite	9 (16%)	2 (5.9%)	0.20		

**Weeks between tests**	8 (7; 10)	8 (7; 8)	0.13		

**Comorbidities n (%)**					
Asthma	8 (14%)	2 (5.9%)	0.31		
Hypertension	4 (7.0%)	3 (8.8%)	1.00		
Hyperthyroidism	3 (5.3%)	1 (2.9%)	1.00		
Cardiac disease	3 (5.3%)	2 (5.9%)	1.00		
Hyperlipidaemia	1 (1.8%)	2 (5.9%)	0.55		
Anxiety/depression	4 (7.0%)	1 (2.9%)	0.65		

**Months from illness to test**	6.6 ± 3.8	n/a			

**Severity of illness n (%)**					
Mild	15 (26%)	n/a			
Moderate	36 (63%)	n/a			
Severe	6 (11%)	n/a			

**Lung Function**					
FVC (l)	4.8±1.0	5.0±1.0	0.20	−0.7–0.2	−0.28
FEV_1_ (l)	3.9±0.8	4.1±0.8	0.22	−0.5–0.1	−0.25
FEV_1_/FVC (%)	81.6±5.3	81.0±5.3	0.55	−1.6–3.0	0.13
PEF (l·min^−1^)	8.6±2.5	9.1±2.1	0.28	−1.6–0.5	−0.24
MVV (l·min^−1^)	135.2±27.8	142.21±27.0	0.24	−19.8–4.9	−0.25
MIP (cmH_2_O)	102.0±25.9	107.88±21.8	0.24	−16.7–4.3	−0.26

**Cardiopulmonary exercise testing (CPET)**					
VO_2_peak (ml·kg·min^−1^)	39.1±8.6	42.0±7.6	0.12	−6.4–0.7	−0.34
VO_2_peak% ref	126.6±33.7	128.1±26.3	0.82	−15.0–11.9	−0.05
VE/VCO_2_ slope	32.0±3.1	31.4±2.4	0.30	−0.6–1.9	0.23
y-intercept (l·min^−1^)	1.1±3.5	1.0±2.7	0.89	−1.3–1.5	0.03
VO_2_ at VT (ml·min^−1^)	65.7±10.6	72.6±10.6	0.004	−11.4–−2.3	−0.65
RER at max	1.2±0.6	1.1±0.04	0.05	0–0.05	0.43
VE max (l·min^−1^)	108.1±25.5	115.4±26.6	0.20	−18.4–3.8	−0.28
VE/MVV	80.3±12.6	81.5±14.0	0.67	−6.9–4.4	−0.09
HR max (bpm)	172.8±10.3	174.1±9.7	0.57	−5.6–3.1	−0.12
Max O_2_-pulse (ml·bpm^−1^)	16.4±3.9	18.1±4.6	0.07	−3.5–0.1	−0.40
Drop in SpO_2_ %	7.5 (5;11)	6 (5;8)	0.17	−2–0	0.39
RPE	19 (18;20)	19 (18;20)	0.29	0–1	0
Max Workload (W·kg^−1^)	4.6 (3.9;5.2)	5.21 (5.2;6.2)	0.05	0–1	0.71

95% Confidence Interval and Effect size as applicable. We assume variants based on the elapsed time since onset of illness: omicron <8 weeks, beta 3–6 months, delta >7 months. NIH Symptom classification: Mild: fatigue; malaise; headache; myalgia; ageusia/anosmia; cough; fever; diarrhoea; Moderate: dyspnoea with SpO_2_ >94%; Severe: dyspnoea with SpO_2_ <94%. Data expressed as mean±SD, or median (interquartile range) as applicable. BMI, body mass index; SD, standard deviation; FVC (1), forced vital capacity; FEV_1_ (l), forced expiratory volume in one second; PEF, peak expiratory flow; MVV, maximum voluntary ventilation; VO2peak, maximum volume of oxygen in millilitres consumed, VE, minute ventilation, HR, heart rate; MIP, maximal inspiratory pressure; RER, respiratory exchange ratio; RPE, rating of perceived exertion; VT, ventilatory threshold

**Table 3:** The difference between COVID-19 and N-COVID-19 groups: lung function and CPET from baseline test (1) to follow-up (2)

Parameter	COVID-19 (2)-(1) (n=52)	N-COVID-19 (2)-(1) (n=26)	p value	95% CI	Effect size

**Lung Function**					

FVC (l)	0.06 (−0.04; 0.22)	0.01±0.18	0.60	−0.11–0.7	0.27
FEV_1_ (l)	−0.01±0.24	−0.06±0.17	0.41	−0.06–0.15	0.20
FEV_1_/FVC (%)	−0.89±4.69	−1.01 (−2.75; 0.91)	0.68	−1.65–1.16	0.02
PEF (l·min^−1^)	0.41±1.85	0.25±1.34	0.70	−0.66–0.97	0.09
MVV (l·min^−1^)	−0.40± 8.5	−2±6.07	0.41	5.23–4.9	0.20
MIP (cmH_2_O)	5.12±17.19	−3.46±13.15	0.03	0.94–16.22	0.54

**Cardiopulmonary exercise testing (CPET)**					

VO_2_peak (ml·kg·min^−1^)	1.75±4.31	0.05±3.77	0.09	−0.27–3.69	0.41
VO_2_peak% ref	5.8±14.7	2.8 (−6.09; 6.30)	0.33	−8.9–2.27	0.23
VE/VCO_2_ slope	−0.17±2.39	0.13±1.98	0.58	−1.39–0.78	−0.13
y-intercept (l·min^−1^)	0.5±2.79	0.23±1.66	0.65	−0.91–1.46	−0.11
VO_2_ at VT (ml·min^−1^)	−0.01 (−0.04; 0.03)	0.02 (−0.01; 0.06)	0.05	0.03–0.05	0.58
RER at max	2.50±13.11	−3.08±14.26	0.09	−0.88–12.29	0.41
VEmax (l·min^−1^)	3.35±16.19	1.65±14.16	0.65	−5.75–9.13	0.11
VE/MVV	2.76±10.64	2.57±9.28	0.94	−4.69–5.08	0.02
HRmax (bpm)	−0.06±9.29	0.04±5.30	0.96	−4.02–3.82	−0.01
MaxO_2_-pulse (ml·bpm^−1^)	0.70±2.26	−0.04±1.79	0.14	−0.26–1.76	0.12
Drop in SpO_2_%	−1.0 (−4.3; 0)	0 (−2; 2)	0.31	−1.0–3.0	−0.32
RPE	0 (−1.0; 1.0)	0 (−0.6; 1.0)	0.61	0–1	0
Max Workload (W·kg^−1^)	0 (0; 1.25)	0 (0; 0.2)	0.27	−0.06–0	0

Data expressed as mean±SD, or median (interquartile range) as applicable. FVC (1), forced vital capacity; FEV_1_ (l), forced expiratory volume in one second; PEF, peak expiratory flow; MVV, maximum voluntary ventilation; VO2peak, maximum volume of oxygen in millilitres consumed, VE, minute ventilation, HR, heart rate; MIP, maximal inspiratory pressure; RER, respiratory exchange ratio; RPE, rating of perceived exertion; VT, ventilatory threshold

**Table 4:** Pulmonary and extra-pulmonary limitations to exercise capacity in COVID-19 and N-COVID-19 groups at baseline test

Parameter indicating limitation	Condition	COVID-19 n=57	N-COVID-19 n=34	p-value	OR	95% CI
VO_2_peak <100% ref applicable to athletes^19^	No limitation	43 (75%)	28 (82%)	0.44	1.5	0.52–4.42
	With limitation	14 (25%)	6 (18%)			
FEV_1_/FVC <80%^26^	No limitation	36 (63%)	22 (65%)	0.88	1.1	0.44–2.58
	With limitation	21 (37%)	12 (35%)			
VE/MVV 70–80%^19^	No limitation	16 (28%)	9 (27%)	0.87	0.9	0.36–2.4
	With limitation	41 (72%)	25 (74%)			
Drop in SpO_2_ >10% in athletes^14^	No limitation	47 (83%)	33 (97%)	0.05	7.0	0.86–57.53
	With limitation	10 (18%)	1 (3%)			
VE/VCO_2_ slope >34^19^	No limitation	44 (77%)	28 (82%)	0.56	1.4	0.47–4.05
	With limitation	13 (23%)	6 (17%)			
MIP <85% ref	No limitation	40 (70%)	30 (88%)	0.07	3.2	1.15–12.2
	With limitation	17 (30%)	4 (12%)			
MIP <85% ref and abnormal breathing reserve	No limitation	45 (79%)	32 (94%)	0.07	4.3	0.89–20.39
	With limitation	12 (21%)	2 (6%)			
Abnormal breathing reserve and abnormal resting lung function with normal VO_2_peak (80%–120%) = true pulmonary limitation^30^	No limitation	53 (93%)	30 (88%)	0.47	0.6	0.13–2.43
	With limitation	4 (7%)	4 (12%)			
Abnormal breathing reserve with normal resting spirometry and supranormal VO_2_peak (>120%) = physiological limitation^30^	No limitation	42 (74%)	23 (67.6%)	0.54	0.8	0.3–1.89
	With limitation	15 (26%)	11 (32.4%)			
Max O_2_-pulse^17^	No limitation	46 (80.7%)	28 (82.4%)	0.85	1.1	0.37–3.35
	With limitation	11 (19.3%)	6 (17.6%)			
VT <60%VO_2_peak	No limitation	43 (75.4%)	32 (94.1%)	0.03	5.2	1.11–24.56
	With limitation	14 (24.6%)	2 (5.9%)			

ref, reference value; OR, odds ratio; FVC (1), forced vital capacity; FEV_1_ (l), forced expiratory volume in one second; PEF, MVV, maximum voluntary ventilation; VO_2_peak, maximum volume of oxygen in millilitres consumed, VE, minute ventilation; MIP, maximal inspiratory pressure; VT, ventilatory threshold
