# Supplementary material for: Cardiorespiratory fitness response to endurance training in athletes post-COVID-19 compared to unaffected athletes
Source: S Afr J Sports Med. 2024 Jan 15;36(1):v36i1a18872. doi: 10.17159/2078-516X/2024/v36i1a18872 (PMC11629943; doi:10.17159/2078-516X/2024/v36i1a18872)
Supplement: Supplementary file 1 [file 2078-516X-36-v36i1a18872-s001.pdf]

Cardiorespiratory fitness response to endurance training in athletes post-COVID-19 compared to unaffected athletes

Supplementary Table 1. Comparison of lung function and CPET between the first test (1) and follow up test (2) in the COVID-19 group.

Parameter	COVID-19 (1) (n=57)	COVID-19 (2) (n=52)	*p*-value	Effect size
**Lung function**				
FVC (l)	4.8 ± 1.0	4.4 (3.9; 5.7)	0.60	0.29
FEV_1 (l)	3.9 ± 0.8	3.9 ± 0.9	0.73	0.05
FEV1/FVC	81.6 ± 5.3	80.7 ± 4.8	0.18	0.19
PEF ($l·min^{-1}$)	8.6 ± 2.5	9.0 ± 2.7	0.11	-0.22
MVV ($l·min^{-1}$)	135.2 ± 27.8	133.4 ± 34.4	0.98	0.22
MIP (cmH2O)	102.0 ± 25.9	105.5 ± 24.0	0.04	-0.38
**Cardiopulmonary exercise testing (CPET)**				
VO_2peak ($ml·kg·min^{-1}$)	39.1 ± 8.6	41.2 ± 8.5	0.005	-0.41
VO_2peak % ref ($ml·kg·min^{-1}$)	126.6 ± 33.7	134.0 ± 31.0	0.006	-0.40
VE/VCO_2 slope	32.0 ± 3.1	32.0 ± 2.7	0.61	0.07
y-intercept ($l·min^{-1}$)	1.09 ± 3.5	1.4 ± 2.7	0.15	0.27
RER at max	1.2 ± 0.6	1.2 (1.1; 1.2)	0.42	0.02
VO_2 at VT ($ml·min^{-1}$)	65.7 ± 10.6	68.3 ± 9.3	0.18	-0.19
VE max ($l·min^{-1}$)	108.1 ± 25.5	112.8 ± 28.7	0.14	-0.21
VE/MVV	80.3 ± 12.6	83.9 ± 13.2	0.075	-0.26
HR max (bpm)	172.8 ± 10.3	172.9 ± 7.8	0.96	0.00
O_2-pulse ($ml·bpm^{-1}$)	16.4 ± 3.9	17.2 ± 4.0	0.029	-0.31
Drop in SpO_2 %	7.3 ± 3.2	5 (4.0; 8.3)	0.07	0.73
RPE	18.8 ± 1.0	19 (19; 19	0.66	-0.93
Workload ($W·kg^{-1}$)	4.8 ± 1.1	5.2 (5.1; 6.0)	<0.001	-0.50

Data expressed as mean ± standard deviation or (median; interquartile range) as applicable and determined using the T-test for paired samples or Wilcoxon signed rank test. RPE: Rate of perceived; FVC (1), forced vital capacity; FEV_1 (l), forced expiratory volume in one second; PEF, peak expiratory flow; MVV, maximum voluntary ventilation; VO_2peak, maximum volume of oxygen in millilitres consumed, VE, minute ventilation, HR, heart rate; MIP, maximal inspiratory pressure; RER, respiratory exchange ratio; VT, ventilatory threshold

Supplementary Table 2. Comparison of lung function between the first test (1) and follow-up test (2) in the N-COVID-19 group.

Parameter	N-COVID-19 (1) (n=34)	N-COVID-19 (2) (n=26)	*p*-value	Effect size
**Lung function**				
FVC (l)	5.0 ± 1.0	5.0 ± 1.0	0.75	-0.06
FEV_1 (l)	4.1 ± 0.8	4.0 ± 0.8	0.11	0.32
FEV_1/FVC	81.0 ± 5.3	81.0 (68.2; 78.3)	0.06	-0.009
PEF ($l·min^{-1}$)	9.1 ± 2.1	9.6 ± 2.1	0.33	-0.19
MVV ($l·min^{-1}$)	142.2 ± 27.0	139.7 ± 26.6	0.11	0.32
MIP (cmH_2O)	107.9 ± 21.8	104.7 ± 23.7	0.19	0.26
**CPET**				
VO_2peak ($ml·kg·min^{-1}$)	42.0 ± 7.6	42.4 ± 7.6	0.95	-0.012
VO_2peak % ref ($ml·kg·min^{-1}$)	128.1 ± 26.3	124.8 (112.3; 132.2)	0.74	0.15
VE/VCO_2 slope – measured	31.4 ± 2.4	31.6 ± 2.6	0.75	-0.06
y-intercept ($l·min^{-1}$)	0.99 ± 2.7	1.3 ± 2.3	0.51	0.21
RER at max	1.1 ± 0.04	1.2 (1.1; 1.2)	0.00	-0.49
VO_2 at VT ($ml·min^{-1}$)	72.6 ± 10.6	70.0 ± 9.1	0.28	0.22
VE max ($l·min^{-1}$)	115.4 ± 26.6	116.2 ± 30.1	0.56	-0.12
VE/MVV	81.5 ± 14.0	83.8 ± 17.8	0.72	-0.28
HR max (bpm)	174.1 ± 9.7	174.3 ± 9.1	0.97	-0.007
O_2-pulse ($ml·bpm^{-1}$)	18.1 ± 4.6	17.9 ± 4.6	0.89	0.025
Drop in SpO_2 %	6 (5;8)	5 (4;10)	0.92	0.32
RPE	19 (18;20)	19 (19;20)	0.33	0
Workload ($W·kg^{-1}$)	5.2 (5.2;6.1)	5.2 (5.2;6.3)	0.23	-0.034

Data expressed as mean ± standard deviation or (median; interquartile range) as applicable and determined using the T-test for paired samples or Wilcoxon signed rank test. RPE: Rate of perceived; FVC (1), forced vital capacity; FEV_1 (l), forced expiratory volume in one second; PEF, peak expiratory flow; MVV, maximum voluntary ventilation; VO_2peak, maximum volume of oxygen in millilitres consumed, VE, minute ventilation, HR, heart rate; MIP, maximal inspiratory pressure; RER, respiratory exchange ratio; VT, ventilatory threshold

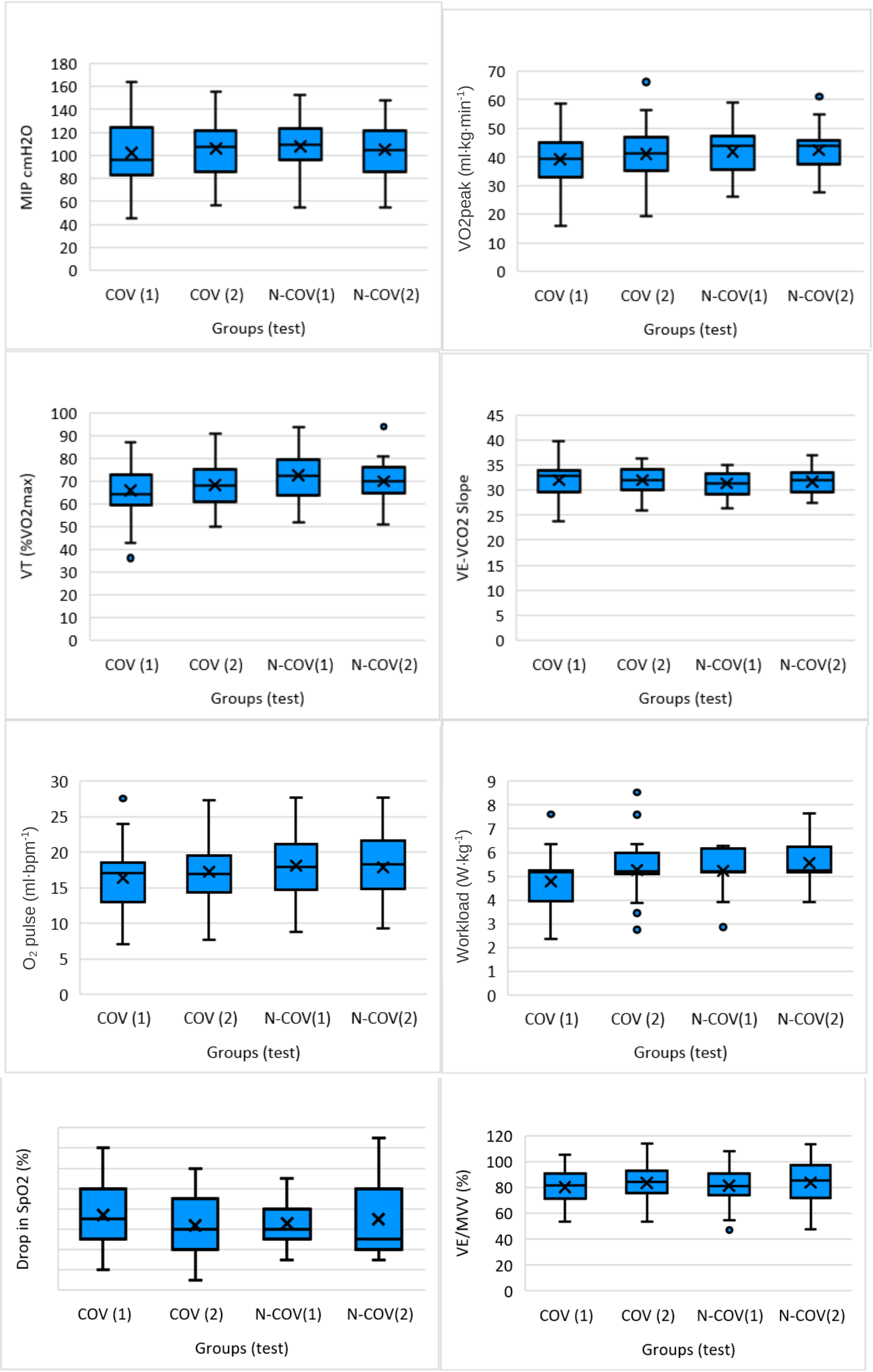

***Supplementary Figure 1**. Distribution of physiological parameters across the four tests: baseline (1), follow-up (2), COVID-19 group (COV), N-COVID-19 group (N-COV)*
